# Host nectin-1 is required for efficient *Chlamydia trachomatis* serovar E development

**DOI:** 10.3389/fcimb.2014.00158

**Published:** 2014-11-06

**Authors:** Jennifer V. Hall, Jingru Sun, Jessica Slade, Jennifer Kintner, Marissa Bambino, Judy Whittimore, Robert V. Schoborg

**Affiliations:** ^1^Department of Biomedical Sciences, Quillen College of Medicine, East Tennessee State UniversityJohnson City, TN, USA; ^2^Center for Inflammation, Infectious Disease and Immunity, Quillen College of Medicine, East Tennessee State UniversityJohnson City, TN, USA; ^3^College of Medical Sciences, Washington State UniversitySpokane, WA, USA

**Keywords:** *Chlamydia trachomatis*, herpes simplex virus, co-infection, Nectin-1, persistence, persistent chlamydiae, chlamydial stress response

## Abstract

Interaction of Herpes Simplex Virus (HSV) glycoprotein D (gD) with the host cell surface during *Chlamydia trachomatis*/HSV co-infection stimulates chlamydiae to become persistent. During viral entry, gD interacts with one of 4 host co-receptors: HVEM (herpes virus entry mediator), nectin-1, nectin-2 and 3-*O*-sulfated heparan sulfate. HVEM and nectin-1 are high-affinity entry receptors for both HSV-1 and HSV-2. Nectin-2 mediates HSV-2 entry but is inactive for HSV-1, while 3-*O*-sulfated heparan sulfate facilitates HSV-1, but not HSV-2, entry. Western blot and RT-PCR analyses demonstrate that HeLa and HEC-1B cells express nectin-1 and nectin-2, but not HVEM. Because both HSV-1 and HSV-2 trigger persistence, these data suggest that nectin-1 is the most likely co-receptor involved. Co-infections with nectin-1 specific HSV-1 mutants stimulate chlamydial persistence, as evidenced by aberrant body (AB) formation and decreased production of elementary bodies (EBs). These data indicate that nectin-1 is involved in viral-induced chlamydial persistence. However, inhibition of signal transduction molecules associated with HSV attachment and entry does not rescue EB production during *C. trachomatis*/HSV-2 co-infection. HSV attachment also does not activate Cdc42 in HeLa cells, as would be expected with viral stimulated activation of nectin-1 signaling. Additionally, immunofluorescence assays confirm that HSV infection decreases nectin-1 expression. Together, these observations suggest that gD binding-induced loss of nectin-1 signaling negatively influences chlamydial growth. Chlamydial infection studies in nectin-1 knockdown (NKD) HeLa cell lines support this hypothesis. In NKD cells, chlamydial inclusions are smaller in size, contain ABs, and produce significantly fewer infectious EBs compared to *C. trachomatis* infection in control HeLa cells. Overall, the current study indicates that the actions of host molecule, nectin-1, are required for successful *C. trachomatis* development.

## Introduction

According to the Centers for Disease Control and Prevention, sexually transmitted diseases (STDs) are among the most common infections in the United States. Approximately 19 million new cases of STDs occur each year in the United States, almost half of them among people ages 15–24 (Weinstock et al., 2004; Centers for Disease Control and Prevention, 2012). Two of the most commonly reported STD agents in the United States are *Chlamydia trachomatis* (serovars D-K; 2.8 million new cases/year) and Herpes Simplex Virus (primarily HSV-2; 200,000–500,000 new cases/year) (Butler, 1997; Centers for Disease Control and Prevention, 2012).

*C. trachomatis* is a Gram-negative, obligate intracellular bacterium. Long-term *C. trachomatis* genital tract infections are often chronic and asymptomatic, resulting in ascending infections and complications, such as epididymitis, prostatitis, endometritis, salpingitis, ectopic pregnancy and infertility (Darville et al., 2000). All chlamydiae share a unique biphasic developmental cycle, alternating between two morphologically distinct forms. The extracellular infectious form (the elementary body or EB, 0.3 μm diameter) attaches to and enters mucosal epithelial cells via receptor mediated endocytosis (Wyrick, 2000). Following the fusion of EB-containing endosomes, EBs develop into larger (1 μm diameter), metabolically active but non-infectious reticulate bodies (RBs). Using ATP and metabolites from the host cell, RBs grow and divide within an enlarged endosomal sac, the inclusion. After 8–12 rounds of replication, the RBs mature into infectious EBs, which are released from the host cell (Wyrick, 2000).

When developing chlamydiae are exposed to unfavorable environmental conditions, they deviate from the normal developmental cycle into a state termed persistence or, alternatively, the chlamydial stress response. Persistent or stressed chlamydiae are characterized by formation of aberrantly enlarged, viable but non-infectious chlamydial RBs (Hogan et al., 2004; Schoborg, 2011). Persistent chlamydiae continue to synthesize unprocessed 16S rRNA and replicate chromosomes but fail to divide (Gerard et al., 1998, 2001). Known persistence inducers include IFN-γ, TNF-α and penicillin-exposure as well as amino acid, glucose and iron deprivation (Beatty et al., 1994; Raulston, 1997; Darville et al., 2000; Gerard et al., 2001). Notably, the persistent chlamydiae can re-enter and complete the normal developmental cycle once the “inducer” is removed. Several studies suggest that under appropriate circumstances, chlamydial persistence may occur *in vivo* in humans (Patton et al., 1994; Fortenberry et al., 1999; Dean et al., 2000; Bragina et al., 2001; Gerard et al., 2001). Recently, persistence induction *in vivo* has been definitively demonstrated using a murine model of amoxicillin-induced *C. muridarum* persistence (Phillips Campbell et al., 2012).

Herpes simplex virus types 1 and 2 (HSV-1 and HSV-2) are members of the viral family *Herpesviridae*. While HSV-2 is the major causative agent of genital herpes, HSV-1 also causes genital infections with a similar disease presentation to that of HSV-2. HSV infection can cause serious diseases in humans, such as keratitis and meningitis, although most genital HSV infections are clinically mild (Roizman and Knipe, 2001). The HSV virion has a large double-stranded DNA genome (~150 kbp). The genome is packed within a capsid shell, which is in turn coated with a protein layer, the tegument, and an envelope composed of lipids and more than a dozen viral proteins and glycoproteins (Spear and Longnecker, 2003). Entry of HSV into cells is initiated when the virion binds to cell surface receptor heparan sulfate using the viral envelope proteins gC and/or gB. After the initial binding, the viral gD envelope glycoprotein interacts with one of 4 cellular co-receptors, including herpes virus entry mediator (HVEM), nectin-1, nectin-2, or 3-O sulfated heparan sulfate (3-O-S-HS) (Spear, 2004). Glycoprotein D binding to any one of these co-receptors facilitates the fusion of the viral envelope with the host cell plasma membrane, followed by release of the capsid/tegument structure into the cytoplasm. Subsequently, the capsid is transported to the host nucleus, where the viral DNA genome is transcribed. New virions are assembled in the nucleus and egress the host cell by vesicular transport (Roizman and Knipe, 2001).

A number of studies have shown that *C. trachomatis* and HSV-2 co-infections occur *in vivo*. Both pathogens have been simultaneously isolated from the genital tract of women afflicted with endometritis and salpingitis or cystitis (Paavonen et al., 1985; Tait et al., 1985). In addition, several groups have also established HSV/chlamydial co-infection in cell culture. These observations indicate that HSV-2 co-infection alters chlamydial development (Pontefract et al., 1989; Chiarini et al., 1996; Superti et al., 2001) by inducing chlamydial persistence (Deka et al., 2006). Induction of *C. trachomatis* serovar E persistence by HSV is neither host cell type or virus strain specific, nor are *de novo* host/viral protein synthesis and productive HSV replication required (Deka et al., 2007). Additionally, HSV glycoprotein D (gD) interaction with host cell surface is sufficient to induce chlamydial persistence (Vanover et al., 2010). It has also been demonstrated that herpes virus-induced oxidative stress inhibits *C. trachomatis* L2 development, causing the bacteria to become persistent (Prusty et al., 2012). Because gD/co-receptor binding is a prerequisite for HSV entry into host cells, we hypothesized that HSV interaction with a known co-receptor is sufficient to alter the chlamydial developmental cycle by causing alterations in cell signaling and/or inducing oxidative stress within the host cell.

## Materials and methods

### Cells, viruses and chlamydiae

Chinese hamster ovary (CHO) cell lines including CHO-C8 (with the pcDNA3 vector alone), CHO-HVEM, CHO-nectin-1 and CHO-nectin-2 were kind gifts from Dr. Patricia Spear, Northwestern University. Additional cell lines used in the study are HeLa cells, a cervical adenocarcinoma epithelial cell line (ATCC No. CCL2), and HEC-1B cells, an endometrial epithelial cell line (ATCC No. HTB-113). Wild type HSV strains HSV-2 333 and HSV-1 KOS were obtained from Dr. Mary K. Howett (Drexel University) and Dr. Udayasankar Kumaraguru (East Tennessee State University), respectively. The parental stain HSV-1 KOS/FRT-gD (expressing wild type gD), mutant HSV-1 strains, HSV-1 KOS/FRT-gD_G43P_, HSV-1 KOS/FRT-gD_Q27P_, and HSV-1 KOS/FRT-gD_A3C/Y38C_ and the HSV-2/Gal mutant (HSV-2/β g) were obtained from Dr. Patricia Spear (Northwestern University) (Yoon and Spear, 2004; Taylor et al., 2007). The parental and mutant HSV-1 strains and HSV-2/β g express β-galactosidase activity upon host cell entry (Yoon and Spear, 2004). *C. trachomatis* serovar E/UW-5/CX (CtE) was originally obtained from Dr. S. P. Wang and Dr. C. C. Kuo (University of Washington) and *C. muridarum* strain Wiess (Cm) was obtained from Dr. Kyle Ramsey (Midwestern University).

### Co-infection experimental design

Co-infections were performed as previously described by Deka et al. (2006). Host cells were divided into four groups for mock-infection, chlamydial-infection, HSV-infection, and both *Chlamydia*/HSV double infection. First, monolayers were mock- or *Chlamydia*-infected with a dilution of crude EB stock calculated to infect >80% of the cells. Following a 1 h attachment/entry period, all cells were refed with fresh culture medium (Minimal essential medium/10% FBS; Life Technologies) and incubated for 6 (*C. muridarum*) or 24 h (*C. trachomatis*) at 35°C. Monolayers were then mock-infected or infected with 10 MOI HSV-2, HSV-2/β g, HSV-1 or HSV-1 mutant strains for 1 h, refed with fresh culture medium or medium containing the reducing agent, N-acetyl cysteine (NAC, 5 mM) and incubated for 30 min or 20 h at 35°C. Mock-infected cells were treated similarly except they were exposed to either 2SPG (0.2 M sucrose, 6 mM NaH_2_PO_4_, 15 mM Na_2_HPO_4_, 5 mM l-glutamine, pH 7.2; mock chlamydial infection) or growth medium (mock viral infection). In a subset of experiments, host signaling pathway inhibitors were added to the infected samples. At 12 h post *C. trachomatis* infection, cultures were exposed to PBS or a protein kinase B/Akt inhibitor (Akt, 25 uM IMG-2007, Imgenex). In replicate samples, either DMSO (diluent) or chemical inhibitors for phosphoinositide-3 kinase (PI3K, 100 uM LY294002, Cell Signaling), Janus kinase (JAK, 15 nM #420097, Calbiochem, Inc.) or c-Jun N-terminal kinase (JNK, 10 uM SP600125, Sigma) were added to the culture medium individually or as a combined inhibitor cocktail at 23 h post chlamydial infection. Inhibitors were maintained in the culture medium throughout HSV-2 infection.

### SDS-PAGE and western blotting

Monolayers of host cells were lysed and denatured as previously described (Deka et al., 2006). The Western blot assays were conducted as described by Sun et al. (2008). The total protein concentration in cell lysates was normalized by analysis of a SYPRO Ruby stain (Bio-Rad) using a G-box (Bio-Rad) and SynGene software. Primary antibodies were anti-nectin-1 CK6 (sc-21722, Santa Cruz Biotechnology), anti-β-actin (MAB1501, Chemicon), anti-nectin-2 (AF2229, R&D systems), anti-HVEM (N-19) (sc-7766, Santa Cruz Biotechnology), anti-phospho-Akt (9271, Cell Signaling), anti-phospho-JAK (3331, Cell Signaling) anti-phospho-JNK (9251, Cell Signaling), anti-phospho-PI3K (4228, Cell Signaling) and anti-focal adhesion kinase c20 (FAK, sc-558, Santa Cruz). Primary antibody binding was detected with corresponding horseradish peroxidase-conjugated secondary antibodies and visualized using SuperSignal West Pico reagent (Pierce). Densitometry analysis was performed using a FX phosphorimager and Quantity One V2.5.0 software (Bio-Rad) or with a G-box and SynGene software (Biorad). To control for small variations in cell number and gel loading between sample lanes, the nectin-1 quantity in each sample was normalized to the amount of β-actin protein detected in that same lane (Sun et al., 2008).

### RNA isolation, reverse transcription and RT-PCR

Total RNA was isolated from experimental samples using the RNeasy Mini (Qiagen) kit and RT-PCR was performed as described previously (Deka et al., 2006). Experimental template cDNAs were diluted from 1/10–1/1000 in double-distilled H_2_O and synthetic control DNA targets were diluted from 10–0.01 pg/ml, ensuring that each reaction was quantified in the linear amplification range. Specific primers and synthetic control DNA targets for HVEM and nectin-2 were designed using Vector NTI Advance V10 (Invitrogen) and listed in Table S1. Nectin-1α, -1β, and -1γ specific primers and their oligonucleotide amplification controls were as previously described (Sun et al., 2008). Most reactions were performed using the following cycling conditions (unless otherwise indicated): 94°C, 1 min; 60°C, 1 min; 72°C, 1 min for 35 cycles. The resulting PCR products were electrophoresed and quantified as previously described (Sun et al., 2008).

### Establishment and infection of nectin-1 knockdown stable cell lines

The shRNA SureSilencing system (Qiagen) was used according to manufacturer's protocol. Briefly, a control vector containing a scrambled sequence and a vector containing a nectin-1 target sequence were amplified in *E. coli* cultured in Luria broth. Vector plasmids were purified and transfected into subconfluent HeLa cell monolayers in 60 mm dishes using D'fect transfection reagent (Dharmacon). To isolate stable cell lines, cells were maintained in Earle's Medium containing 10% heat-inactivated FBS, gentamicin and 800 mg/ml hygromycin B. Colonies were harvested by trypsinizing colonies confined in sterile cloning cylinders and pipetting them from the 60 mm dishes into a 12 well plate. Cells were further expanded into control (Ctl) and nectin-1 knockdown (NKD) stable cell lines. Nectin-1 knockdown was confirmed by PCR using nectin-1α specific primers (data not shown) and Western blot analysis using anti-nectin-1 antibody CK6 (1:100; Santa Cruz) normalized to total protein as determined by SYPRO ruby staining (Bio-Rad) and analyzed with GeneTools software (Syngene). For infection, cells were plated at 1.8 x 10^5^ cells/well in triplicate wells of 24 well plates in antibiotic free media for 24 h. Cells were infected with CtE for 1 h at 35°C. Inocula were removed and cells were refed with antibiotic free media then harvested at 48 hpi.

### Percent infectivity assay

Triplicate Ctl or NKD monolayers on glass coverslips were infected with a dilution of crude CtE EB stock calculated to infect approximately 20% of the cells, such that accurate quantification of inclusion number per cell nuclei could be calculated. After 1 h, inocula were removed and monolayers were refed with antibiotic free medium. At 48 hpi, monolayers were fixed and permeabilized with 1 ml cold methanol for 20 min. Coverslips were stained with Pathfinder anti-chlamydial MOMP stain (Bio-Rad), counterstained with DAPI and mounted on glass slides. The number of inclusions and cell nuclei in 10 fields/coverslip was determined at 400 × magnification with an Axiovert S100 (Zeiss) microscope and Axiovert imaging software.

### Chlamydial EB titration analysis

Chlamydial titrations were conducted as previously described (Deka et al., 2006) using Pathfinder anti-chlamydial stain (Bio-Rad) to stain chlamydial inclusions formed from subpassaged EBs. The number of inclusion-forming units (IFU) in the undiluted inoculum was derived from triplicate counts and expressed as IFU/ml.

### Measurement of chlamydial inclusion size

*C. trachomatis*-infected Ctl and NKD cultures were harvested at 48 hpi by methanol fixation and stained using Pathfinder anti-chlamydial stain (Bio-Rad). Inclusions were visualized with an Axiovert S100 (Zeiss) microscope. The area of 16 random inclusions from 2 reticule fields (320 × magnification) in replicate samples was measured using Axiovert imaging software. The area of each inclusion in pixels was used to determine the average inclusion size in CtE-infected Ctl and NKD cell lines.

### Transmission electron microscopy

*Chlamydia*-infected or *Chlamydia*/HSV co-infected HeLa cells were processed for high-contrast TEM as described (Wyrick et al., 1994). Counter-stained gold thin sections were examined using a Tecnai 10 (FEI) transmission electron microscope operating at 60–80 kV.

### β-galactosidase (β-gal) assay for HSV entry

Many of the HSV strains used in this study are engineered to express β-gal after host cell entry, which allows expression of this enzyme to be used as an indirect marker of host cell entry (Yoon and Spear, 2004; Taylor et al., 2007). Therefore, we monitored HSV virion entry into host cells by X-gal staining after infection with β-gal expressing HSV strains, as described previously (Montgomery et al., 1996; Vanover et al., 2010).

### Glutathione assay

At 30 min and 20 h post viral infection (pvi), replicate CtE- and CtE/HSV-2-infected monolyaers cultivated in standard tissue culture medium were harvested for quantification of glutathione (GSH) and the oxidized disulfide dimer GSSG using a Glutathione Assay (Cayman Chemical) according to the manufacturer's instructions. Absorbance was measured using a Turner Modulas microplate reader.

### Nectin immunofluorescence assay

Replicate HeLa cell monolayers were mock or HSV-2-infected as described above. Monolayers were formaldehyde fixed and permeabliazed with NP-40 immediately prior to viral infection (T-1) or 6 h (T6) and 20 h (T20) post HSV-2 infection. Nectin-1 expression in the fixed monolayers was evaluated by use of anti-nectin-1 CK6 primary antibody (sc-21722, Santa Cruz Biotechnology) and Alexa Fluor 488 dye (Life Technologies). Nectin-1 expression was visualized at 100 × magnification with an Axiovert S100 (Zeiss) microscope and Axiovert imaging software.

### Cdc42 activation assay

Activation of Cdc42 was measured in mock or HSV-1-infected HeLa monolayers at 15, 30, and 60 min pvi using the Active Cdc42 Pull-Down and Detection Kit (Thermo Scientific) according to the manufacturer's instructions.

### Statistical analyses

Statistical analyses were performed using Microsoft Excel. A two-sample *t*-test for independent samples was used for comparison of means; *p* ≤ 0.05 were considered significant. Unless otherwise stated all experiments were performed at least 3 times independently with triplicate biological replicates in each experiment. The results reported are the mean ± standard error of the mean (s.e.m.).

## Results

### HeLa and HEC-1B cells express nectin-1 and nectin-2 but not HVEM

Previous data from our laboratory demonstrated that HSV co-infection-induced chlamydial persistence occurs in both HeLa and HEC-1B cells (Deka et al., 2007). To begin elucidating which host co-receptor/s is/are required for HSV to interfere with chlamydial development, we examined HSV co-receptor expression on HeLa and HEC-1B cells. Duplicate HeLa or HEC-1B cell lysates were subjected to SDS-PAGE and Western blot analysis using antibodies against HVEM, nectin-1, nectin-2 and β-actin. Equal concentrations of CHO-HVEM, CHO-nectin-1 and CHO-nectin-2 cell lysates were also subjected to Western blot analysis as positive controls. CHO-C8 cells express cell surface HS but lack any known HSV co-receptors. HSV-1 KOS binds to these cells, but does not enter, even at multiplicities (10-100 MOI) that infect HeLa cells with 100% efficiency (Johnson et al., 1989). The co-receptor-deficient CHO cell lines CHO-HVEM, CHO-nectin-1 and CHO-nectin-2 express recombinant HVEM, nectin-1 and nectin-2, respectively, rendering them permissive for HSV-1 KOS entry and productive replication (Yoon and Spear, 2004). As shown in Figure S1A, both HeLa and HEC-1B cells express nectin-1 and nectin-2, but not HVEM. CHO-HVEM, CHO-nectin-1 and CHO-nectin-2 cell lines express a large amount of HVEM, nectin-1 or nectin-2 respectively, as expected (Figure S1A). CHO-nectin-1′ and CHO-nectin-2′ depict the same protein bands as shown in the CHO-nectin-1 and CHO-nectin-2 gels, using a shorter exposure time (Figure S1A).

In addition, total RNA from HeLa, HEC-1B or CHO-HVEM cells was isolated and subjected to RT-PCR using specific primers for HVEM. In each experiment, a four log dilution series of synthetic control DNA was used to generate standard curves for amplification. Experimental samples were only quantified if they fell within the linear range of the PCR. All amplimers were the expected size (Figure S1B) and the identity of each was confirmed by DNA sequencing (data not shown). Amplification products were not observed in template-negative samples (Figure S1B, lane 6) or in RT(-) controls (data not shown). As shown in Figure S1B, CHO-HVEM cells express HVEM transcripts; however, HVEM transcripts were not detected in HeLa or HEC-1B samples (Figure S1B) even when the PCR was extended to 38 cycles (data not shown). Similarly, total RNA from HeLa, HEC-1B, CHO-nectin-1,or CHO-nectin-2 cells was isolated and subjected to RT-PCR using specific primers for nectin-1α, -1β, and -1γ (Figure S1C) and/or nectin-2 (Figure S1D). As shown in Figure S1, both HeLa and HEC-1B cells express nectin-1 and nectin-2 transcripts, although to a lesser degree than CHO-nectin-1 or CHO-nectin-2 cells. Finally, Sun et al. demonstrated that HeLa cells express nectin-1α protein and mRNA transcripts of all three isoforms (Nectin-1α, -1β, and -1γ) by RT-PCR (Sun et al., 2008).

**Figure 1:**
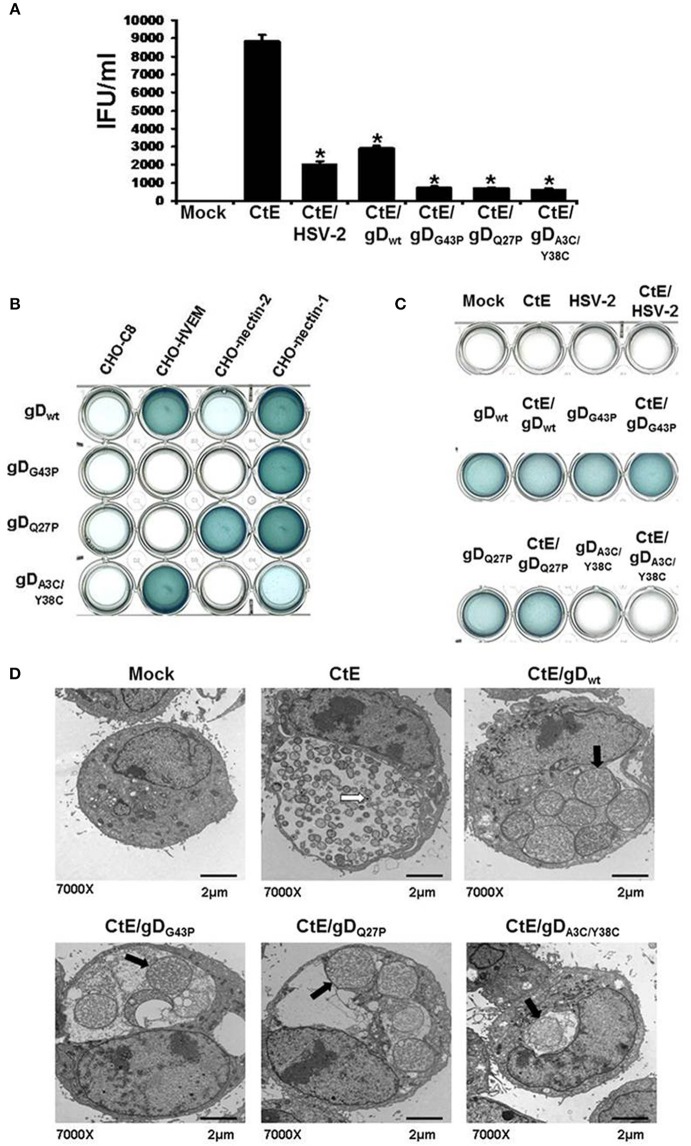
**HSV co-infection-induced chlamydial persistence signal requires viral attachment/entry via nectin-1. (A)** HeLa cells were either mock-, singly-, or co-infected with CtE and various wild type (HSV-2 and HSV-1 gD_wt_) or mutant viruses (gD_G43P_, gD_Q27P_, and gD_A3C/Y38C_). Cells were collected 20 h pvi and processed for chlamydial EB titration analyses. Results are expressed as the mean ±s.e.m. of three biological replicates. A single asterisk (^*^) indicates significant difference (*p* ≤ 0.05) compared to CtE. **(B)** CHO-C8, CHO-HVEM, CHO-nectin-1 and CHO-nectin-2, were infected with HSV-1 gD_wt_, gD_G43P_, gD_Q27P_, and gD_A3C/Y38C_ for 6 h. Viral entry was analyzed by β-galactosidase assay. **(C)** HeLa cells were either mock, singly or co-infected with *C. trachomatis* and HSV-2, HSV-1 gD_wt_, gD_G43P_, gD_Q27P_, or gD_A3C/Y38C_ for 6 h. Viral entry was analyzed by β-galactosidase assay. **(D)** HeLa cells were either mock-, CtE-infected or co-infected with CtE and HSV-2, HSV-1 gD_wt_, gD_G43P_, gD_Q27P_, or gD_A3C/Y38C_. Cells were harvested for TEM analyses. White arrows on electron micrographs indicate EBs and black arrows indicate abnormally enlarged RBs characteristic of persistence (i.e., abberent bodies or AB).

Finally, we used β-galactosidase (β-gal) assays to assess nectin-1, nectin-2 and/or HVEM mediated HSV entry in HeLa cells. HeLa monolayers were infected with wild-type HSV-1 or HSV-1 gD mutants that exhibit specific binding efficiencies to HSV co-receptors, as previously shown by Yoon and Spear (2004). The parental and mutant HSV-1 strains express β-gal upon host cell entry (Yoon and Spear, 2004). The parental strain, HSV-1 KOS/FRT-gD_wt_ (gD_wt_), expresses wild type gD protein and enters host cells via HVEM, nectin-1 and 3-O-S-HS with high/moderate efficiency and via nectin-2 with very low efficiency. HSV-1 KOS/FRT-gD_G43P_(gD_G43P_) can only enter using nectin-1, whereas HSV-1 KOS/FRT-gD_Q27P_ (gD_Q27P_) uses both nectin-1 and nectin-2 equally well. Neither gD_G43P_ nor gD_Q27P_ use HVEM or 3-O-S-HS, even when infections are performed at 200 MOI. Both HVEM and 3-O-S-HS facilitate entry of HSV-1 KOS/FRT-gD_A3C/Y38C_(gD_A3C/Y38C_), but nectin 1 and 2 do not (Yoon and Spear, 2004). As shown in Figure 1C, β-gal activity was present in HeLa cells infected with gD_wt_, gD_Q27P_, and gD_G43P_. However, gD_A3C/Y38C_ did not enter HeLa cells, as evidenced by the lack of β-gal activity, confirming that HeLa cells express little or no HVEM co-receptor (Figure S1E).

Collectively, these data demonstrate that both HeLa and HEC-1B cells express nectin-1 and nectin-2, but do not express HVEM. In addition, it has been shown that anti-HVEM antibodies had marginal effects on HSV-1 entry into HeLa cells, suggesting that HVEM is probably not the major receptor mediating HSV entry into HeLa cells (Montgomery et al., 1996). On the other hand, previous studies have demonstrated that both HSV-1 and HSV-2 trigger chlamydial persistence (Deka et al., 2007) and both HSVs use HVEM and nectin-1 equally well (Spear, 2004). Moreover, nectin-2 mediates HSV-2 entry with high efficiency but is essentially inactive for HSV-1, while 3-O-S-HS facilitates HSV-1, but not HSV-2 entry (Spear, 2004). We have previously demonstrated that interaction of non-clustered soluble HSV-2 gD:Fc fusion proteins with HeLa cells had little effect on chlamydial development, whereas IgG-preclustered gD:Fc significantly decreased infectious EB production (Vanover et al., 2010). Notably, the clustering of nectins is required for nectin trans-interaction induced down-stream cell signaling pathways (Ogita and Takai, 2006). Therefore, our data suggest that binding of clustered gD:Fc to host cell surface nectins leads to clustering of nectin molecules and activation of host cell signaling events. Thus, evidence suggests that, of the known HSV co-receptors, nectin-1 is the most likely candidate involved in HSV co-infection-induced chlamydial persistence.

### HSV co-infection-induced chlamydial persistence requires nectin-1-mediated viral entry

Our published data suggest that HSV gD/co-receptor interaction halts the chlamydial developmental cycle by altering host cell signal transduction and, hence, downstream physiologic functions (Vanover et al., 2010). To further investigate whether this phenomenon requires viral attachment/entry via nectin-1, we performed co-infections using HSV-1 mutants with altered co-receptor specificity. HeLa cells were either mock, singly, or co-infected with *C. trachomatis* (CtE) and various wild type (HSV-2 and gD_wt_) or mutant HSV-1 strains (gD_G43P_, gD_Q27P_, and gD_A3C/Y38C_). HSV-2 co-infection was used as a positive control for chlamydial persistence induction. Infected monolayers were collected 20 h pvi and processed for chlamydial EB titration. As shown in Figure 1A, HSV-2, gD_wt_ and the three HSV-1 mutants all significantly reduced chlamydial EB production. Since we have found that HeLa cells do not express HVEM and gD_A3C/Y38C_ has been reported to be HVEM/3-O-S-HS specific (Yoon and Spear, 2004), it was unexpected to observe that the double mutant repressed chlamydial infectivity.

To further examine this observation, we performed β-gal assays to check the specific entry phenotypes of gD_wt_, gD_G43P_, gD_Q27P_, and gD_A3C/Y38C_ in CHO-C8, CHO-HVEM, CHO-nectin-1 and CHO-nectin-2 cells. As expected, no β-gal activity was observed in in CHO-C8 cells, since CHO-C8 cells lack any known HSV co-receptors (Figure 1B). β-gal activity was readily apparent in CHO-HVEM and CHO-nectin-1 cultures, but was much lower in CHO-nectin-2 cells infected with gD_wt_. These results were expected because nectin-2 supports HSV-2 entry but is nearly inactive for entry of HSV-1 (Spear, 2004). As expected, gD_Q27P_ entered both CHO-nectin-1 and CHO-nectin-2 cells, while only CHO-nectin-1 cells were infected with gD_G43P_. Significant β-gal activity was also observed in gD_A3C/Y38C_ infected CHO-HVEM cells, which was unsurprising because this mutant has been observed to be HVEM specific (Yoon and Spear, 2004). However, we also observe low but detectable β-gal activity in gD_A3C/Y38C_ infected CHO-nectin-1 cells, which indicates that despite previously published reports (Yoon and Spear, 2004), gD_A3C/Y38C_ can attach to and enter nectin-1 expressing CHO cells, albeit to a lesser degree than CHO-HVEM cells. Thus, overall, the only co-receptor that is utilized by all three mutants is nectin-1.

We also tested the ability of gD_wt_, gD_G43P_, gD_Q27P_, and gD_A3C/Y38C_ to enter HeLa cells. As shown in Figure 1C, no β-gal activity is observed in mock, CtE, HSV-2, and CtE /HSV-2-infected HeLa cells. As expected, the wild type (gD_wt_) and the two mutants (gD_G43P_ and gD_Q27P_), both of which have high affinity for nectin-1, enter HeLa cells with high efficiency. However, the double mutant gD_A3C/Y38C_does not have detectable entry activity for HeLa cells in this assay. This is not surprising since this assay is relatively insensitive and nectin-1 expression on HeLa cells is much lower than on CHO-nectin-1 cells, as evidenced by the data shown in Figure S1A.

To confirm the results from Figure 1A, we conducted transmission electron microscopy (TEM), because persistent forms of *C. trachomatis* have a characteristic electron microscopic appearance (Matsumoto and Manire, 1970; Beatty et al., 1994). Mock, CtE, and CtE/gD_wt_, CtE/gD_G43P_, CtE/gD_Q27P_, or CtE/gD_A3C/Y38C_- infected cultures were collected at 20 h pvi and processed for TEM analysis. Electron micrographs demonstrated that in CtE singly-infected HeLa cells, EBs were present and RBs appeared normal (Figure 1D). In contrast, in cultures co-infected with CtE and gD_wt_, gD_G43P_, gD_Q27P_, or gD_A3C/Y38C_, EBs were absent from chlamydial inclusions and chlamydiae exhibited aberrant RB morphology as well as increased membrane blebs, characteristic of persistence (Figure 1D). Collectively, these data are consistent with the supposition that nectin-1 is required for HSV co-infection-induced chlamydial persistence.

### Inhibition of cellular signaling pathways activated during HSV attachment and entry does not restore EB production during CtE/HSV co-infection

**Figure 2:**
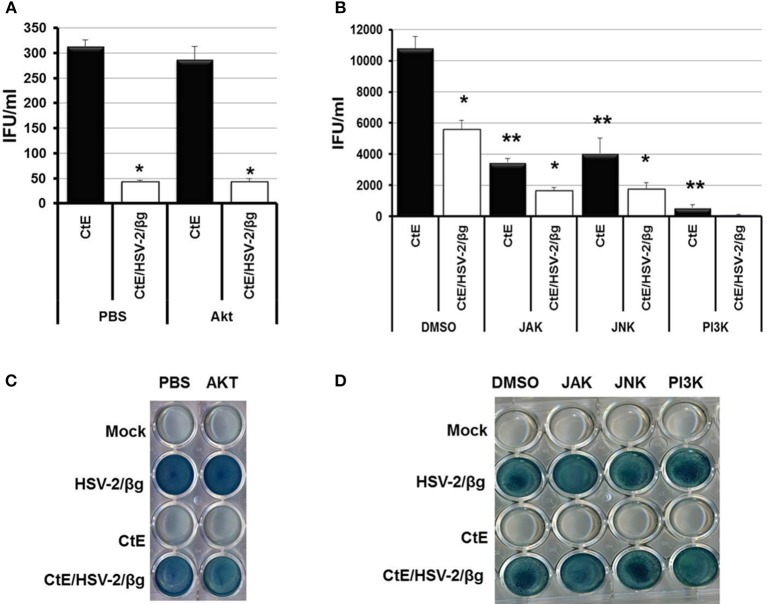
**Inhibition of Akt, JAK, JNK, or PI3K during CtE/HSV-2/βg co-infection does not restore EB production**. Replicate HeLa monolayers were mock-, CtE-, HSV-2/β g-, and CtE/HSV-2/β g -infected. **(A)** PBS or an Akt inhibitor was added to the culture medium 12 h post CtE infection and remained in the cultures throughout HSV infection. **(B)** DMSO or a JAK, JNK, or PI3K inhibitor was added to the culture medium 23 h post CtE infection and remained in the cultures throughout HSV infection. Cultures were harvested at 20 h pvi. EB production in CtE and CtE/HSV-2/β g cultures was determined by EB titration analysis. A single asterisk (^*^) indicates significant difference (*p* = 0.05) between CtE and CtE/HSV-2/β g within an experimental condition. Results are expressed as the mean ±s.e.m. of three biological replicates. Double asterisks (^**^) indicate significant difference (*p* = 0.05) between DMSO- and JAK-, JNK-, or PI3K-exposed CtE-singly infected samples. **(C,D)** Viral entry was analyzed by β-galactosidase assay.

Hoppe et al. demonstrated that attachment and entry of HSV-1 into MDCKII cells stimulates cellular signaling through activation of the small Rho-like GTPase, Cdc42 (Hoppe et al., 2006). However, Cdc42 activation was not observed in HeLa cells following HSV-1 infection (Figure S2A). Attachment of HSV to co-receptors and the subsequent entry of virions into host cells triggers host cell NF-κ B, PI3K/Akt, JAK/STAT, and JNK/Src-responsive pathways (Chen and Silverstein, 1992; Amici et al., 2006; Hoppe et al., 2006; MacLeod and Minson, 2010). Western blot analysis also demonstrated increased phosphorylation of Akt, JAK, JNK, and PI3K 6 h pvi in CtE/HSV-2 co-infected lysates compared to CtE lysates (Figure S2B). Thus, we wanted to determine if cellular signaling pathways activated during HSV attachment and entry are responsible for the observed decrease in EB production during CtE/HSV-2 co-infection. In these experiments we used a strain of HSV-2 that expresses β-galactosidase (HSV-2/β g) to ensure that the inhibitors did not negatively affect viral entry (Taylor et al., 2007). Replicate HeLa monolayers were mock, or CtE infected. At 12 h post chlamydial infection, cultures were exposed to either PBS or an Akt inhibitor. In replicate cultures, DMSO or PI3K, JAK, or JNK inhibitors were added to the culture medium individually or combined as an inhibitor cocktail at 23 h post chlamydial infection. At 24 h post chlamydial infection, cultures were infected with HSV-2/β g, so that mock, CtE, HSV-2/β g, and CtE/ HSV-2/β g cultures were generated. Inhibitors were maintained in the culture medium throughout the HSV-2/β g infection. At 20 h pvi, samples were collected for examination of viral entry and EB production. β-gal was expressed in all HSV-2/β g and CtE/HSV-2/β g samples regardless of inhibitor addition, indicating that none of the inhibitors tested reduced HSV-2/β g attachment or entry into host cells (Figures 2C,D). HSV-2/β g co-infection decreased EB production compared to CtE infection alone, in the presence of DMSO, PBS or Akt, PI3K, JAK, or JNK inhibitors, indicating the inhibitors did not reverse the HSV co-infection-induced reduction in infectious EB production (Figures 2A,B). Interestingly, EB production in CtE singly-infected cultures was decreased in the presence of PI3K, JAK, and JNK inhibitors compared to DMSO-exposed controls, indicating that these HSV entry-stimulated pathways are necessary for development of infectious chlamydial progeny (Figure 2B). Furthermore, when CtE-infected cultures were exposed to a cocktail of PI3K/JAK/JNK inhibitors, chlamydial infectivity was completely abolished regardless of HSV co-infection (data not shown). These data suggest that (i) PI3K, JAK, and JNK are important for normal *C. trachomatis* development, and (ii) the stimulation of these pathways during CtE/HSV co-infection is not responsible for HSV-induced chlamydial persistence induction.

### Knockdown of nectin-1 expression decreases EB production

**Figure 3:**
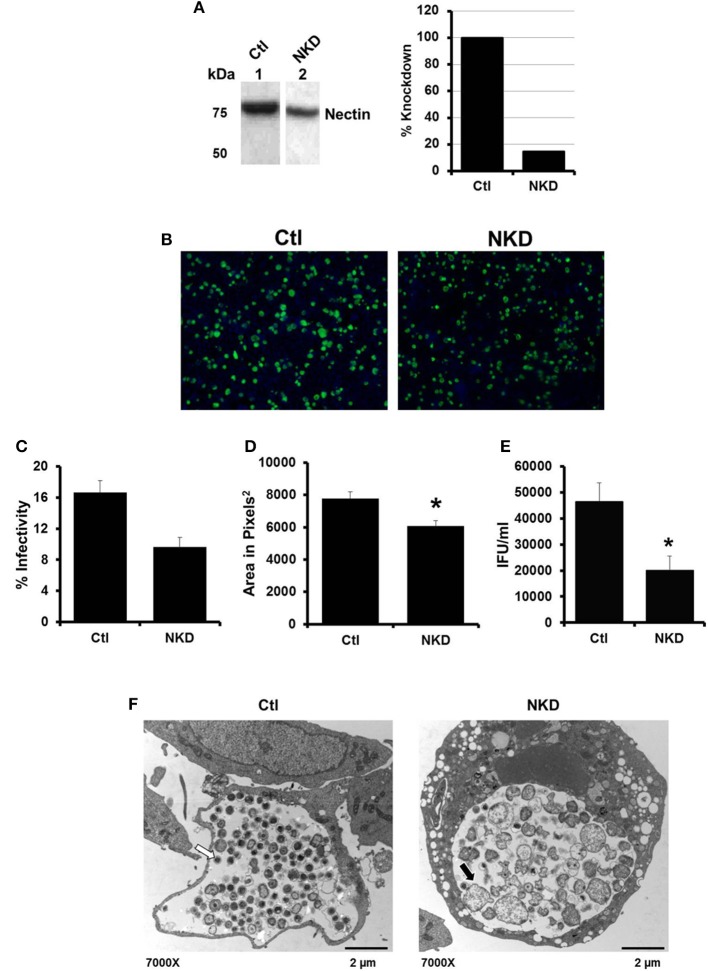
**Production of *C. trachomatis* infectious progeny, but not inclusion formation, is nectin-1 dependent**. **(A)** Nectin-1 accumulation in control (Ctl, lane 1) and nectin-1 knockdown (NKD, lane 2) cell lines was verified by Western blot analysis. Intensity was normalized to total protein intensity detected by SYPRO Ruby staining (data not shown) and analyzed with Gene Tools software (Syngene). Intensity measurements are shown as percent of Ctl. **(B–F)** Replicate cultures of Ctl and NKD cells were infected with CtE and harvested at 48 hpi for various analyses. **(B)** Chlamydial inclusions were stained with Pathfinder anti-chlamydial stain (green). Cell nuclei were counterstained with DAPI (blue). Images were captured at 100 × magnification on a Ziess Axiovert S100 microscope. **(C)** Percent infectivity was calculated by counting the number of inclusions/cell nuclei in 10 random fields per coverslip from triplicate samples. **(D)** Area of 32 random inclusions per triplicate samples was measured using Ziess Axiovision software. **(E)** The production of infectious EB in CtE-infected Ctl and NKD was determined by titration analysis. **(C–E)** Results are expressed as the mean ±s.e.m. of three biological replicates. Significant (*P* ≤ 0.05) difference from CtE-infected Ctl cells is indicated by the asterisk (^*^). **(F)** CtE-infected Ctl and NKD cultures were analyzed by TEM. White arrows on electron micrographs indicate EBs and black arrow indicate ABs.

By 6 h post HSV infection, nectin-1 expression is decreased in HeLa cells (Figure S2C; Krummenacher et al., 2003). Given that inhibition of HSV-activated signaling pathways did not rescue EB production; we hypothesized that the loss of nectin-1 functions subsequent to HSV binding and entry may trigger chlamydial persistence. To test this hypothesis, we examined *C. trachomatis* infection in a nectin-1 knockdown HeLa cell line. Control (Ctl) and nectin-1 knockdown (NKD) cell lines were generated using a scrambled sequence or nectin-1 specific shRNA, respectively. Knockdown of nectin-1 protein was confirmed by Western blot analysis (Figure 3A). Replicate Ctl and NKD monolayers were infected with CtE and harvested at 48 hpi for analysis of inclusion formation, EB production, and chlamydiae morphology by TEM. Both the Ctl and NKD cell lines were successfully infected with CtE (Figure 3B). While not statistically different, there was a slight decrease in percent infectivity in the CtE-infected NKD cells compared to the Ctl cultures (Figure 3C). Interestingly, the inclusion size in the CtE-infected NKD cells was significantly smaller (*p* < 0.05) compared to that in CtE-infected Ctl cultures (Figures 3B,D). Production of infectious EB was also significantly reduced (*p* < 0.005) in NKD cultures compared to the Ctl cells (Figure 3E). Transmission electron micrographs reveal AB formation in NKD cells compared to normal RB and EB formation in the Ctl cell line (Figure 3F, black arrow). Together these data indicate that functional nectin-1 is required for optimal development of chlamydial inclusions and infectious progeny.

### HSV co-infection inhibits *C. muridarum* progeny EB production and stimulates AB formation

**Figure 4:**
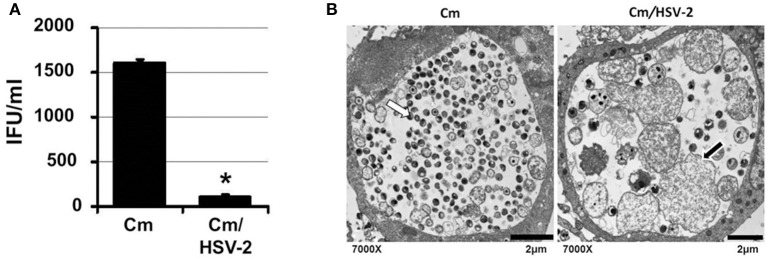
**HSV co-infection disrupts *C. muridarum* development**. HeLa cells were either Cm- or Cm/HSV-2-infected. In co-infected cultures, cells were infected with Cm 6 h before HSV-2 infection. Samples were collected 20 h pvi and processed for chlamydial EB titration analyses **(A)** and TEM analysis **(B)**. White arrows on electron micrographs indicate EBs and black arrows indicate ABs.

*C. muridarum* causes an infection in mice similar to *C. trachomatis* in humans (Everett et al., 1999). As *C. muridarum* (Cm) is a widely used *in vivo* experimental model for chlamydial pathogenesis, we wanted to determine if this species responded to HSV co-infection similarly to *C. trachomatis*. HeLa monolayers were infected with *C. muridarum* (Cm) for 6 h, followed by HSV-2 infection, such that mock, Cm, HSV-2 or Cm/HSV-2-infected cultures were generated. Infected monolayers were collected 20 h pvi and processed for chlamydial EB titration and TEM analysis. Viral co-infection was performed at 6 h post chlamydial infection because Cm has a more rapid developmental cycle compared to CtE. Additionally, we performed co-infections at 6, 12 or 24 h post Cm infection and observed that HSV-2 co-infection at 6 h post Cm infection has the greatest effect on Cm development (data not shown). Compared with Cm singly-infected controls, a significant decrease in the production of infectious EBs occurred in co-infected cells (Figure 4A). In Cm/HSV-2 co-infected samples AB formation was observed (Figure 4B, black arrow). Additionally, inclusions in co-infected cells contained few EBs compared to Cm-infected samples, which exhibited primarily EBs and RBs of normal size and morphology (Figure 4B). These data indicate that HSV co-infection-induced chlamydial persistence is not *Chlamydia* species specific.

### Oxidative stress contributes to CtE/HSV co-infection induced reduction of EB progeny production

**Figure 5:**
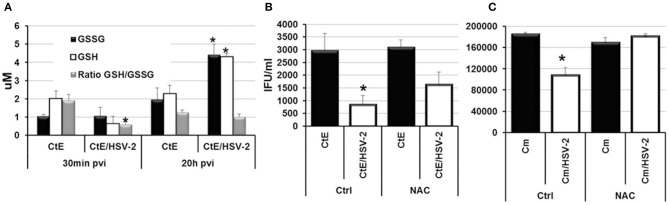
**N-acetyl cysteine exposure during *Chlamdyia*/HSV-2 co-infection rescues EB production during *Chlamydia*/HSV-2 co-infection**. **(A)** Triplicate HeLa monolayers were either CtE- or CtE/HSV-2-infected. Monolayers were harvested at 30 min and 20 h pvi for quantification of GSH and GSSG. **(B)** Triplicate HeLa monolayers were either CtE- or CtE/HSV-2-infected. **(C)** Triplicate HeLa monolayers were either Cm- or Cm/HSV-2-infected. Following HSV-2 infection, cultures were replenished with either standard culture medium (Ctrl) or medium containing NAC (5 mM). Monolayers were harvested at 20 h pvi for EB titration analysis. Results are expressed as the mean ±s.e.m. of three biological replicates. Significant (*P* ≤ 0.05) difference between *Chlamydia* singly-infected and *Chlamydia*/HSV-2 co-infected samples is indicated by the asterisk (^*^).

Recently, it has been demonstrated that Human Herpes Virus-6 (HHV6) co-infection stimulates *C. trachomatis* L2 persistence via increased oxidative stress and decreased levels of reduced glutathione (GSH; Prusty et al., 2012). Prusty et al. also demonstrated that the reducing agent DTT could restore chlamydial infectivity during HSV-1/*C. trachomatis* L2 co-infection, suggesting that oxidative stress during co-infection is a mechanism of persistence induction by both alpha and beta herpes viruses (Prusty et al., 2012). To determine if HSV-2 co-infection induces *C. trachomatis* serovar E persistence in a similar manner, we performed CtE/HSV-2 co-infections in the presence of the reducing agent, N-acetyl cysteine (NAC). Triplicate HeLa monolayers were either mock-, CtE-, HSV-2- or CtE/HSV-2-infected. Following HSV-2 infection, monolayers were replenished with either culture medium (Ctrl) or medium containing NAC (5 mM). At 30 min and 20 h pvi, replicate CtE- and CtE/HSV-2-infected Ctrl monolayers were harvested for quantification of glutathione (GSH) and the oxidized disulfide dimer GSSG. At 20 h pvi, replicate Ctrl and NAC-exposed, mock-, CtE-, HSV-2-, CtE/HSV-2-infected cultures were harvested for analysis of EB production. The GSH/GSSG ratio was significantly decreased in co-infected cultures verses CtE singly-infected samples at 30 min pvi; however, by 20 h pvi the GSH/GSSG ratio was not significantly different between CtE and CtE/HSV-2 samples (Figure 5A), indicating that viral entry transiently increases oxidative stress in co-infected cells, as previously observed (Dickinson and Forman, 2002). In the Ctrl samples, CtE/HSV-2 co-infection significantly decreased EB production compared to CtE singly-infected cultures, as expected. Conversely, NAC exposure partially restored chlamydial infectivity in HSV-2 co-infected cultures (Figure 5B), as observed by Prusty et al. (2012). These data suggest that, by protecting the host cell from oxidative stress, NAC reduces the effect of HSV co-infection on CtE development. Duplicate co-infection experiments in the presence or absence of NAC were also performed using *C. muridarum.* Production of Cm EB was completely restored in co-infected, NAC-exposed cells (Figure 5C). Taken together these data suggest that early events in HSV-2 replication induce oxidative stress in *Chlamydia*/HSV-2-infected host cells contributing to induction of chlamydial persistence during HSV-2 co-infection.

## Discussion

When exposed to certain adverse environmental factors, developing chlamydiae deviate from the normal developmental cycle into a viable but non-replicative state variably termed persistence or chlamydial stress (Hogan et al., 2004; Schoborg, 2011; Bavoil, 2014) and can remain in this state for weeks or months (Galasso and Manire, 1961). While controversial, persistent chlamydial infections have been hypothesized to increase the likelihood that an individual will experience prolonged inflammation of the genital tract, increasing the possibilities for development of severe disease sequelae (Beatty et al., 1994; Wyrick, 2010). Previously, our laboratory established a tissue culture model of *C. trachomatis*/HSV-2 co-infection. Data from this model indicate that HSV attachment, specifically, interactions between HSV gD and host co-receptors, is sufficient to stimulate chlamydiae to become persistent (Deka et al., 2006, 2007; Vanover et al., 2008, 2010).

HSV gD is the main determinant of cell recognition for viral entry. During the initial stages of viral invasion, gD binds to one of its co-receptors: nectin-1, nectin-2, 3-O-S-HS and HVEM, which is followed by viral entry. Nectin-1 and nectin-2 are important cell adhesion molecules of the immunoglobulin super-family and are distributed on fibroblasts and epithelial cells. They are involved in formation and maintenance of adherens junctions and tight junctions (Sakisaka and Takai, 2004). HSV-1 and HSV-2 have specific co-receptor binding affinities. While nectin-2 mediates HSV-2 entry and 3-O-S-HS facilitates HSV-1 entry, both viruses can use nectin-1 and HVEM to enter host cells (Spear, 2004). HSV-1 and HSV-2 can drive developing chlamydiae to enter persistence (Deka et al., 2007), suggesting that gD interactions with either nectin-1 or HVEM could trigger HSV co-infection-induced persistence. Additional data indicate that co-receptors responsible for transmitting the persistence signal must be clustered by gD before chlamydial development can be effected (Vanover et al., 2010). Because nectins must cluster prior to activation of cell signaling (Ogita and Takai, 2006), these data suggest that nectin-1-mediated signaling is involved. Additional evidence indicating that nectin-1 plays a critical role in HSV co-infection-induced chlamydial persistence is two-fold. First, both HeLa and HEC-1B host cell lines support HSV-induced persistence (Deka et al., 2007) and express nectin-1 and nectin-2, but not HVEM. Second, HSV-1 mutants that use nectin-1 as an entry receptor all induce *C. trachomatis* persistence. Surprisingly, we found that the gD_A3C/Y38C_ mutant induces chlamydial persistence, despite the fact that HeLa cells do not express detectable HVEM and the previous observation that gD_A3C/Y38C_ is HVEM/3-O-S-HS specific (Yoon and Spear, 2004). However, in contrast to previous reports, we observed that gD_A3C/Y38C_ enters CHO-HVEM cells with low efficiency. Therefore, the only co-receptor that the parental and three mutant strain share is nectin-1. Together, these results suggest that HSV binding/entry via nectin-1 is required to trigger a subsequent host cellular response, which ultimately restricts chlamydial development.

*Trans*-interaction of nectins activates cellular Cdc42 small G proteins through c-Src. Activated Cdc42 and c-Src then activate Rac small G proteins. Finally, Cdc42 and Rac selectively activate c-Jun N-terminal kinase, but not p38 MAP kinase or extracellular signal-regulated kinase (ERK) (Takai and Nakanishi, 2003; Takai et al., 2003). Activated Cdc42 and Rac ultimately regulate cell-cell adhesion, gene expression and cell polarization (Takai et al., 2003). Interestingly, transient Cdc42 and Rac1 activation is observed in MDCKII canine kidney cells from 15–30 min after HSV-1 infection (Hoppe et al., 2006). Furthermore, HSV and/or gD binding stimulates NF-κ B, PI3K/Akt, JAK/STAT and JNK/Src-responsive pathways (Chen and Silverstein, 1992; Amici et al., 2006; Hoppe et al., 2006; MacLeod and Minson, 2010). We also observed increased phosphorylation of Akt, JAK JNK and PI3K in CtE/HSV-2 co-infected cells compared to CtE-infected cultures. Based upon this information, it is intriguing to envision that the HSV/nectin-1 interaction-activated chlamydial persistence response is mediated through nectin-1-stimulated signaling events. However, in our hands, Cdc42 is not activated in HSV-1-infected HeLa cells. Additionally, inhibition of PI3K, JAK, JNK, or Akt did not rescue *C. trachomatis* EB production during co-infection. In fact, our data indicate that PI3K, JAK, and JNK signaling are crucial to successful EB production at an undetermined point during mid-to-late chlamydial development. Overall, these data suggest that: (i) the co-infection triggered anti-chlamydial response is not mediated through stimulation of nectin-linked host cell signaling; and (ii) the absence of nectin-linked signaling may actually negatively influence chlamydial development.

In HSV-infected cells, binding of gD to nectin-1 alters accumulation of nectin-1 in the plasma membrane, disrupting cellular junctions and nectin-1-associated signaling (Krummenacher et al., 2003). It has even been demonstrated that gD can replace nectin-1 at cell junctions during HSV infection (Krummenacher et al., 2003). Our data confirm that nectin-1 expression is decreased by 6 h pvi in HeLa cells. Chlamydial infection of nectin-1 knockdown cell lines demonstrated that though nectin-1 is not required for chlamydial inclusion development, inclusions are smaller and contain aberrant chlamydiae when host cell nectin-1 expression is reduced. Additionally, nectin-1 is required for maximal production of infectious EB. These data suggest that HSV attachment to nectin-1 triggers chlamydial persistence, at least in part, by interfering with an as yet unidentified nectin-1 function.

It is intriguing to hypothesize that HSV alteration of nectin-1 expression modifies cytoskeletal components that are required for chlamydial inclusion development. *C. trachomatis* interacts with F-actin during EB entry and intracellular growth and F-actin remodeling occurs at the site of EB attachment during chlamydial entry into the host cell (Carabeo et al., 2002). Furthermore, a scaffold of F-actin and intermediate filaments surrounds and may stabilize the developing chlamydial inclusion (Kumar and Valdivia, 2008). *Trans*-interactions of nectins in the formation of adherens junctions also influence actin cytoskeletal rearrangements. Nectin interactions with the F-actin binding protein, afadin, trigger actin reorganization through cellular signaling by Src, Cdc42 and Rac. Our data indicate that Cdc42 is not activated following HSV entry into HeLa cells, suggesting that this pathway is not involved in viral-induced chlamydial persistence. However, following initial nectin interactions, E-cadherins also participate in the formation of junctional complexes and can have effects on F-actin dynamics via a Cdc42 independent activation of Rac (Takai and Nakanishi, 2003; Takai et al., 2003; Miyoshi and Takai, 2008). Thus, it is possible that by changing nectin-1 expression during co-infection, HSV modulates cellular junction regulation of the cytoskeleton in a manner that has negative downstream effects on chlamydial inclusion stability and development.

Prusty et al. demonstrated that imbalanced oxidative stress in HHV6/*C. trachomatis* L2 co-infected cells cause chlamydiae to become persistent (Prusty et al., 2012). Addition of a reducing agent, NAC, to *C. trachomatis* serovar E or *C. muridarum*/HSV-2 co-infected cultures restored chlamydial EB production, indicating that oxidative stress contributes to HSV-2-induced persistence of CtE and Cm. Interestingly, both the data presented above and by Prusty et al. indicate that reducing agents only partially restore EB production in *C. trachomatis* serovar E or L2/Herpes virus co-infected cells (Prusty et al., 2012), suggesting that multiple mechanisms may contribute to viral-induced chlamydial persistence. While both HHV6 and HSV associate with lipid rafts on the surface of host cells, HHV6 uses CD46 as an entry receptor rather than nectin-1 (Tang et al., 2008; Prusty et al., 2012). HHV6 glycoproteins are also not homologous to HSV glycoproteins (Prusty et al., 2012). Thus, it is to be expected that host cell surface interactions with HHV6 and HSV would have different consequences on nectin-1 functions and host signaling events. Interestingly, HSV gD interaction with nectin-1 triggers the release of intracellular calcium stores at the plasma membrane (Cheshenko et al., 2007). Calcium is known to mediate changes in reactive oxygen species accumulation in the eukaryotic cell (Yan et al., 2006). Therefore, it is possible that during HSV attachment and entry, gD interactions with nectin-1 stimulate intracellular calcium release, resulting in transient oxidative stress that ultimately causes chlamydial persistence in co-infected cells.

In conclusion, we have presented data that indicates the HSV co-receptor, nectin-1, contributes to viral-induced chlamydial persistence. The way nectin-1 influences chlamydial growth remains to be elucidated. It is possible that HSV gD/nectin-1 interactions lead to cytoskeletal reorganization or increased oxidative stress in co-infected cells, resulting in an unfavorable environment for chlamydial growth. Alternatively, HSV infection may alter or decrease unknown nectin-1 functions that are crucial for *C. trachomatis* development. Further dissection of nectin-1 functions is warranted, so that we can increase our understanding of the host response to chlamydial infection and provide new and valuable information regarding chlamydia/host cell interactions.

## Author contributions

Jingru Sun made mutant HSV stocks, performed: (i) co-receptor expression assays on HeLa, HEC-1B, and CHO cell lines; and (ii) mutant viral co-infections and all associated titer and TEM studies. Jennifer V. Hall and Marissa Bambino performed phosphoprotein Western blotting. Jessica Slade made the nectin-1 KD cell lines and performed all analyses associated with these cells. Jennifer Kintner and Jennifer V. Hall performed *C. muridarum* co-infections, oxidative stress experiments, and pathway inhibition studies. Jennifer V. Hall, Jingru Sun, Jessica Slade, and Robert V. Schoborg designed the experiments, analyzed data, made the figures and wrote sections of the manuscript. All authors have proof read and approved submission of the manuscript.

### Conflict of interest statement

The authors declare that the research was conducted in the absence of any commercial or financial relationships that could be construed as a potential conflict of interest.
