# Supplementary material for: Host nectin-1 is required for efficient Chlamydia trachomatis serovar E development
Source: Front Cell Infect Microbiol. 2014 Nov 6;4:158. doi: 10.3389/fcimb.2014.00158 (PMC4222120; doi:10.3389/fcimb.2014.00158)
Supplement: Supplementary file 1 [file DataSheet1.PDF]

Supplemental Materials

Supplementary Figures

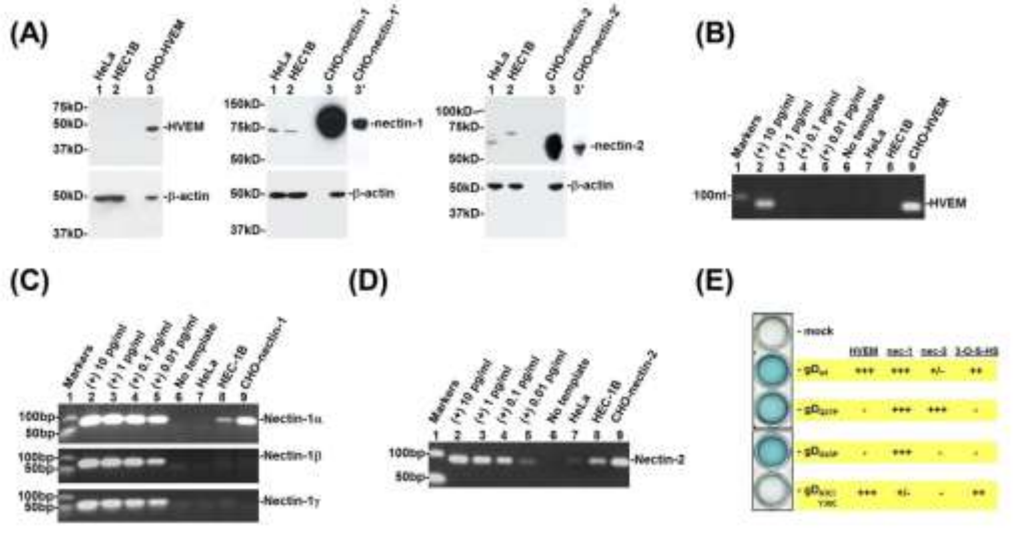

<u>Figure S1:Expression of HVEM, nectin-1 and nectin-2 transcripts in HeLa, HEC-1B and CHO cell lines.</u> **(A)** HeLa, HEC-1B, CHO-HVEM, CHO-nectin-1 or CHO-nectin-2 cell lysates were subjected to SDS-PAGE and Western blotted with either HVEM, nectin-1, nectin-2 or β-actin antibodies. CHO-HVEM, CHO-nectin-1 and CHO-nectin-2 cell lysates were used as positive controls. CHO-nectin-1' and CHO-nectin-2' are shorter exposures of lane 3 in the nectin-1 and nectin-2 blots. **(B)** Total RNA from HeLa, HEC-1B or CHO-HVEM cells was isolated and subjected to RT-PCR using specific primers for HVEM. HVEM amplimers were electrophoresed on an agarose gel and visualized by ethidium bromide staining. A dilution series of synthetic DNA targets was amplified as a positive control. **(C)** Total RNA from HeLa, HEC-1B or CHO-nectin-1 cells was isolated and subjected to RT-PCR analyses using transcript-specific oligonucleotide probes. Nectin-1α, nectin-1β and nectin-1γ amplimers were electrophoresed and photographed; representative photographs are shown. A dilution series of synthetic DNA targets was amplified for each gene as a positive control. The position of DNA size markers are shown to the left of each gel image in base pairs (bp). **(D)** Total RNA from HeLa, HEC-1B or CHO-nectin-2 cells was isolated and subjected to RT-PCR analyses using specific primers for nectin-2. A dilution series of synthetic DNA targets was amplified as a positive control. All amplimers were electrophoresed and photographed; representative photographs are shown. The position of DNA size markers are shown to the left of each gel image in base pairs (bp). **(E)** HeLa cells were infected with parental strain HSV-1 gD_{wt} or the HSV-1 mutants, gD_{G43P}, gD_{Q27P} and $gD_{A3C/Y38C,}$ for 6 hours. β-galactosidase assays were performed as described in the methods. The columns in yellow represent the previously published co-receptor usage for each HSV mutant (Yoon and Spear, 2004). Images were obtained using an Epson Perfection 3200 Photo Scanner and Photoshop Elements Software.

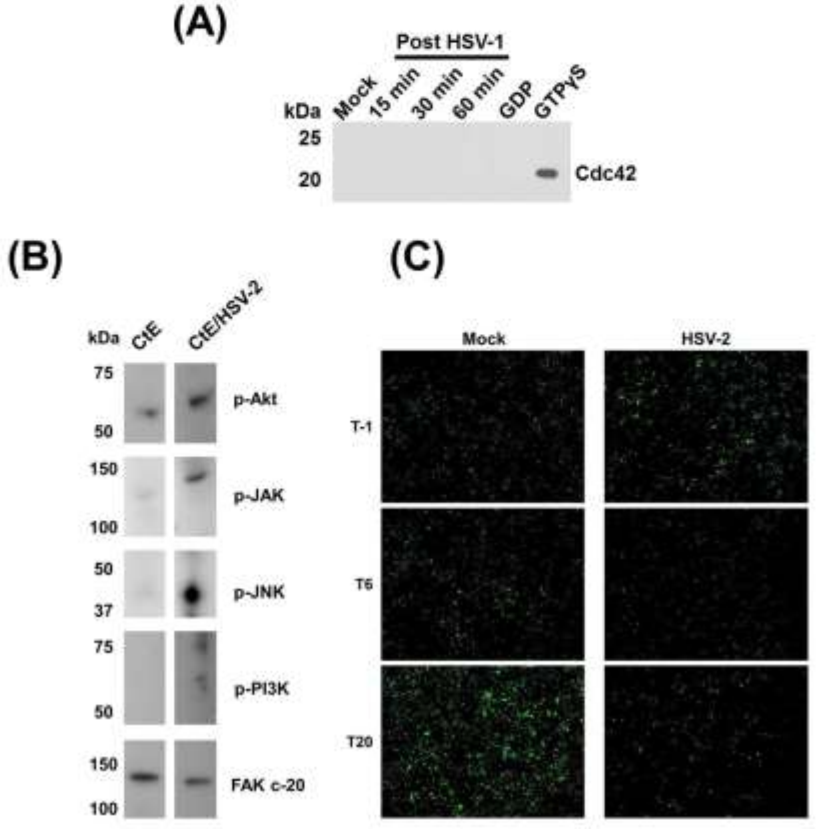

<u>Figure S2:Early changes in nectin-1 expression and host signaling following HSV infection in HeLa Cells.</u> **(A)** Replicate HeLa cell monolayers were mock or HSV-1-infected and collected at 15, 30 or 60 min pvi for analysis of Cdc42 activation by pull-down assay as described in the methods. Mock, negative control (GDP) and positive control (GTPγS) were collected at 60 min pvi. Activated Cdc42 was visualized by Western blot using an anti-Cdc42 mouse monoclonal antibody as directed by the manufacturer. **(B)** HeLa cells were either CtE or CtE/HSV-2 infected and harvested at 6 h pvi for analysis by Western blot as described in the methods using antibodies directed against the phosphorylated host proteins, p-Akt, p-JAK, p-JNK, and p-PI3K. FAK (focal adhesion kinase) was used as loading control. **(C)** Replicate HeLa cell monolayers were mock or HSV-2-infected. Monolayers were harvested immediately prior to viral infection (T-1) or 6 h (T6) and 20 h (T20) post HSV-2 infection for analysis of nectin-1 expression by IFA as described in the methods. Images were taken at 100 x magnification with an Axiovert S100 (Zeiss) microscope and Axiovert imaging software. An image representative of the sample is shown.

Supplementary Table S1. RT-PCR primers and positive control oligonucleotides.

Name	***Forward primer***	***Reverse primer***
Nectin-2	**TCGGAGCACAGCCCACTCAA**	**TTGTGGCTCCAGGGTGCA**
HVEM	**TGTGTGAACCCTGCCCTCCA**	**GCGTTCTCTGTCCTGGAGCA**
Nectin-2 positive control oligonucleotide	**CCTCGGAGCACAGCCCACTCAAGACCCCCTACTTTGATGCTGGCGCCTCATGCACTGAGCAGGAAATGCCTCGATACCATGAGCTGCCCACCTTGGAAGAACGGTCAGGACCCTTGCACCCTGGAGCCACAAGCCTGGGG**	
HVEM positive control oligonucleotide	**CACAGTGTGTGAACCCTGCCCTCCAGGCACCTACATTGCCCACCTCAATGGCCTAAGCAAGTGTCTGCAGTGCCAAATGTGTGACCCAGCCATGGGCCTGCGCGCGAGCCGGAACTGCTCCAGGACAGAGAACGCCGTGT**	
